# Experimentally-driven mathematical modeling to improve combination targeted and cytotoxic therapy for HER2+ breast cancer

**DOI:** 10.1038/s41598-019-49073-5

**Published:** 2019-09-06

**Authors:** Angela M. Jarrett, Alay Shah, Meghan J. Bloom, Matthew T. McKenna, David A. Hormuth, Thomas E. Yankeelov, Anna G. Sorace

**Affiliations:** 1 Institute for Computational Engineering and Sciences, The University of Texas at Austin, Austin, Texas USA; 2 Livestrong Cancer Institutes, The University of Texas at Austin, Austin, Texas USA; 3 Department of Biomedical Engineering, The University of Texas at Austin, Austin, Texas USA; 4 Department of Diagnostic Medicine, The University of Texas at Austin, Austin, Texas USA; 5 Department of Oncology, The University of Texas at Austin, Austin, Texas USA; 6 Department of Biomedical Engineering, Vanderbilt University, Nashville, Tennessee 37232 USA; 7 Department of Radiology, University of Alabama at Birmingham, Birmingham, AL 35209 USA; 8 Department of Biomedical Engineering, University of Alabama at Birmingham, Birmingham, AL 35209 USA; 9 O’Neal Comprehensive Cancer Center, University of Alabama at Birmingham, Birmingham, AL 35209 USA

**Keywords:** Targeted therapies, Computational models, Breast cancer

## Abstract

The goal of this study is to experimentally and computationally investigate combination trastuzumab-paclitaxel therapies and identify potential synergistic effects due to sequencing of the therapies with *in vitro* imaging and mathematical modeling. Longitudinal alterations in cell confluence are reported for an *in vitro* model of BT474 HER2+ breast cancer cells following various dosages and timings of paclitaxel and trastuzumab combination regimens. Results of combination drug regimens are evaluated for drug interaction relationships based on order, timing, and quantity of dose of the drugs. Altering the order of treatments, with the same total therapeutic dose, provided significant changes in overall cell confluence (p < 0.001). Two mathematical models are introduced that are constrained by the *in vitro* data to simulate the tumor cell response to the individual therapies. A collective model merging the two individual drug response models was designed to investigate the potential mechanisms of synergy for paclitaxel-trastuzumab combinations. This collective model shows increased synergy for regimens where trastuzumab is administered prior to paclitaxel and suggests trastuzumab accelerates the cytotoxic effects of paclitaxel. The synergy derived from the model is found to be in agreement with the combination index, where both indicate a spectrum of additive and synergistic interactions between the two drugs dependent on their dose order. The combined *in vitro* results and development of a mathematical model of drug synergy has potential to evaluate and improve standard-of-care combination therapies in cancer.

## Introduction

Combining therapeutic strategies (e.g., targeted, chemo-, radiation-, and immunotherapy, as well as surgery) has become standard-of-care for the treatment of the majority of cancers^1^. In particular, breast cancer patients receive systemic delivery of targeted and cytotoxic drugs (in parallel or sequentially) for various subgroups of receptor positive breast cancers. The additive effects of introducing targeted therapy to cytotoxic treatment have been explored experimentally; however, the synergistic (or even antagonistic) effects of sequencing these regimens in breast cancer have not been systematically investigated. This is likely due to the enormity of such a study as there are nearly limitless combinations of dosing, timing, and ordering of treatments indicated for any given subtype of breast cancer^2^. *In vivo* experiments exploring all possible combinations are impossible, and *in vitro* studies would be time consuming and resource expensive. However, experiment-driven, mathematical modeling could alleviate these challenges by investigating a myriad of combination therapy strategies *in silico* to identify potential treatment regimens for focused *in vivo* and *in vitro* investigations^3^.

One standard combination therapy for the treatment of breast cancers that overexpress the human epidermal growth factor receptor 2 (HER2) is the simultaneous administration of paclitaxel and trastuzumab. HER2 is a transmembrane protein associated with stimulating cellular proliferation. An estimated 25–30% of all breast cancer cases are considered HER2+^4,5^, which is associated with poorer overall prognoses with more aggressive disease compared to HER2- disease. Paclitaxel, a chemotherapy, causes cell death by stabilizing microtubules during mitosis—impeding normal cytokinesis and equal cellular divisions and proliferation^6^. The introduction of trastuzumab, a targeted monoclonal antibody to HER2, two decades ago resulted in a 60% improvement in the median time to disease progression^7^. Trastuzumab binding blocks HER2/neu, preventing receptor dimerization and interfering with intracellular signaling^8^. This obstruction induces cell cycle arrest, inhibits cell proliferation and migration^9–11^, and causes HER2 internalization and subsequent degradation^12,13^. While targeted therapy in combination with systemic cytotoxic therapy has been shown to increase overall survival rates, nearly 26% of patients have recurring disease within 10 years^14^. Since the clinical initiation of trastuzumab, cytotoxic drugs in combination with targeted anti-HER2 therapy, has remained the standard-of-care practice for treatment of primary HER2+ breast cancer. However, *in vivo* preclinical animal data suggests the order, dosing, and timing of combination therapy has yet to be optimized^15^.

**Figure 1:**
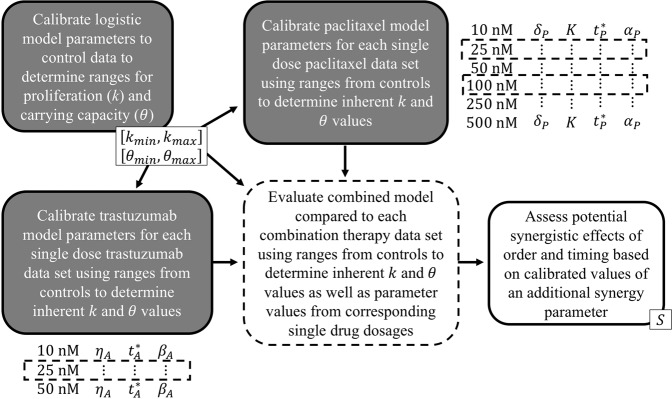
A schematic of the strategy for integrating the experimental evidence with the mathematical models. The logistic model is calibrated to control data to determine ranges for growth and carrying capacity parameters for the cell line. Each single drug model is calibrated to each of the corresponding single drug dose data sets (six sets for paclitaxel, three sets for trastuzumab) using the parameter ranges from the controls to determine the inherent growth and carrying capacity values using the first 24 (drug free) hours for each data set. The combined model utilizes both the ranges generated from controls as well as the parameter values generated by the single drug dose models for the corresponding dosages for the combination drug data sets (indicated with dashed boxes). This combined model is calibrated with an added synergistic parameter to assess potential synergistic effects based on its values for different sequences and dosages of combination therapy.

In this manuscript, we present time-resolved microscopy data that captures changes in *in vitro* cell confluence in response to combination paclitaxel and trastuzumab therapy. The experimental results of the combined regimens are evaluated for synergism versus additive or antagonistic relationships based on order, timing, and quantity of dose of the two drugs. Mathematical models motivated from and constrained by single treatment *in vitro* data to simulate and predict the tumor cell response are then derived. Finally, a collective model merging the two individual drug response models is subsequently designed to reveal potential synergistic and non-synergistic (additive and antagonistic) effects between trastuzumab and paclitaxel due to dosage and timing of the therapies. For a graphical depiction summarizing the integrated experimental-mathematical approach presented here, see Fig. 1.

## Methods

### Cell culture

BT474 HER2+ breast cancer cells were grown in 10% fetal bovine serum, penicillin streptomycin, plasmocin, fungizone, and insulin to 80–90% confluency. Cells were plated in 96-well plates with an initial seeding density of 3.5 × 10^4^ cells per well in 100 *μ*L of media and imaged with an Incucyte Zoom (Essen Bioscience, Ann Arbor, MI) to estimate longitudinal changes in cell confluence at a temporal resolution of 3 hours with phase contrast time resolved microscopy over four days. Cells were allowed to grow freely for the first 24 hours (day 0), before initiating treatment (day 1) with either single or combination therapies. Single agent groups were treated with trastuzumab (Herceptin, Genetech, San Francisco, CA) (10, 25, and 50 *μ*g/mL) or paclitaxel (Teva, United Kingdom) (10, 25, 50, 100, 250, and 500 nM) for a 24 hour exposure time. Additional groups were treated with both drugs in which the order and timing of the therapies was varied. For two different paclitaxel doses (25 nM and 100 nM) in combination with trastuzumab (25 *μ*g/mL), cellular confluence was recorded. Please see Table 1 for a list of all treatment groups. Eight replicates were collected for each treatment condition.

**Table 1:** Summary of the drug combinations of paclitaxel and trastuzumab administered.

Regimen	Paclitaxel Dosage	Trastuzumab Dosage	Timetable
Paclitaxel prior to trastuzumab	25 nM	25 *μ*g/mL	Paclitaxel applied day 1 for 24 hours, trastuzumab applied day 2 for 24 hours
	100 nM		
Trastuzumab prior to paclitaxel	25 nM	25 *μ*g/mL	Trastuzumab applied day 1 for 24 hours, paclitaxel applied day 2 for 24 hours
	100 nM		
Paclitaxel and trastuzumab applied simultaneoulsy	25 nM	25 *μ*g/mL	Paclitaxel and trastuzumab applied day 1 for 24 hours
	100 nM		

### Mathematical models

Mathematical models were developed to describe the response of tumor cells to each of the single agent therapeutic regimens. The models are ordinary differential equations (ODEs), which describe how the tumor cell number changes as a function of time and drug concentration. For the trastuzumab drug response model there are two equations with five total free parameters:

1 $$\frac{dT}{dt}=k{\textstyle (}1-f({A}_{b},t){\textstyle )}{\textstyle (}1-\frac{T}{\theta \,}{\textstyle )}T$$

where *T* is the number of tumor cells, *k* and *θ* are the tumor cell growth rate and carrying capacity, respectively, and

$$f({A}_{b},t)={\textstyle \{}\begin{array}{cc}{\eta }_{A}{A}_{b}, & t > {t}_{A}^{\ast }\\ 0, & t\le {t}_{A}^{\ast }\end{array}$$

represents the reduced proliferation due to bound antibody (*A*_*b*_) with ${\eta }_{A}$ representing the antiproliferative effect per amount of bound trastuzumab. Eq. (1) represents the changes in cell number per time. Note that for control data (i.e., no trastuzumab applied), the function *f*(*A*_*b*_, *t*) is zero, and the model becomes the standard logistic equation for *T*. When trastuzumab is applied, the rate of change of *A*_*b*_ is given by Eq. (2):

2 $$\frac{d{A}_{b}}{dt}={\textstyle \{}\begin{array}{cc}{\beta }_{A}({A}_{f}-{A}_{b})(T\cdot HER{2}_{exp}-{A}_{b}), & {A}_{f} > 0\\ 0, & {\rm{o}}{\rm{t}}{\rm{h}}{\rm{e}}{\rm{r}}{\rm{w}}{\rm{i}}{\rm{s}}{\rm{e}}\end{array}$$

where *A*_*f*_ is the amount of free antibody; per the number of available receptors, ${\beta }_{A}$ is the trastuzumab binding rate, and *HER2*_*exp*_ is the average HER2 expression per tumor cell. This model assumes that only one trastuzumab antibody binds to each receptor. With *A*_*b*_ > 0,  *f*(*A*_*b*_, *t*) becomes non-zero with the antiproliferative effect ${\eta }_{A}$ for all times $t\, > \,{t}_{A}^{\ast }$, where ${t}_{A}^{\ast }$ represents the delay observed between the time of trastuzumab administration until the disruption of intracellular signaling. Please see Table 2 for a list of all variables and parameters, as well as their symbols and how they are assigned.

**Table 2:** Variables and parameters of the mathematical models. Default or median values for the parameters are given across all calibration sets; a more detailed list for each of the calibrations of these values can be found in the supplementary materials.

Variables	Description	Units	
*T*	Number of tumor cells	cells	
*A*_*b*_	Number of bound trastuzumab antibodies	molecules	
*P*_*i*_	Amount of internalized paclitaxel	*p*mol	
*A*_*f*_	Number of free trastuzumab antibodies	molecules	
*P*_*f*_	Amount of free paclitaxel	*p*mol	
**Parameters**	**Description**	**Units**	**Median/Default Value**
*k*	Tumor cell growth rate (calibrated)	1/*t*	0.83
*η*_*A*_	Antiproliferative effect per amount of bound trastuzumab antibody (calibrated)	1/*A*_*b*_	2.51
${t}_{A}^{\ast }$	Delay for bound trastuzumab effect due to intracellular signaling (calibrated)	*t*	1.93
*θ*	Tumor cell carrying capacity (calibrated)	cells	0.67
*δ*_*P*_	Carrying capacity reduction amount per amount of internalized paclitaxel (calibrated)	cells/*P*_*i*_	0.23 × 10^2^
*γ*_*P*_	Rate of decay for initial paclitaxel toxicity (calibrated)	1/*t*	6.35
${t}_{P}^{\ast }$	Time to paclitaxel toxic effect decrease (calibrated)	*t*	1.48
*S*	Drug synergy parameter (calibrated)	unitless	1
*β*_*A*_	Binding rate of trastuzumab antibody per available HER2 receptors (calibrated)	1/(receptors · *t*)	8.78
*HER2*_*exp*_	Average HER2 expression per tumor cell (assigned)	Receptors/cell	1.94 × 10^6^
*α*_*P*_	Paclitaxel internalization rate (calibrated)	1/(cell · *t*)	0.68

For the paclitaxel drug response model there are two equations with six total free parameters:

3 $$\frac{dT}{dt}=k{\textstyle (}1-\frac{T}{\theta -h({P}_{i},t)}{\textstyle )}T$$

where *T* is the number of tumor cells, *k* and *θ* are the tumor cell growth rate and carrying capacity, respectively, and

$$h({P}_{i},t)={\delta }_{P}{\textstyle (}{e}^{-{\gamma }_{P}(t-{t}_{P}^{\ast })}+1{\textstyle )}{P}_{i}$$

represents drug induced death due to internalized paclitaxel. Here, equation (3) represents the changes in cell number per time. Again, note that for control data (no drugs applied), the function *h*(*P*_*i*_, *t*) is zero, and the model becomes the logistic equation. When paclitaxel is applied, the rate of change of *P*_*i*_ is given by Eq. (4):

4 $$\frac{d{P}_{i}}{dt}={\textstyle \{}\begin{array}{cc}{\alpha }_{P}({P}_{f}-{P}_{i})T, & {P}_{f} > 0\\ 0, & {\rm{o}}{\rm{t}}{\rm{h}}{\rm{e}}{\rm{r}}{\rm{w}}{\rm{i}}{\rm{s}}{\rm{e}}\end{array}$$

where *P*_*f*_ is the amount of free paclitaxel with an uptake rate of ${\alpha }_{P}$ per the number of tumor cells (*T*). With internalized paclitaxel (*P*_*i*_ > 0), the function *h*(*P*_*i*_, *t*) becomes non-zero with the carrying capacity reduction effect ${\delta }_{P}$—similar to other modeling studies that modified the carrying capacity due to paclitaxel treatment^16^. The expression ${e}^{-{\gamma }_{P}(t-{t}_{P}^{\ast })}$ represents the immediate toxic effect due to paclitaxel treatment, where ${\gamma }_{P}$ is the rate of decay of this toxicity effect, and ${t}_{P}^{\ast }$ is the time at which the toxic effect becomes less than one and decreases to zero as time progresses. See Table 2 for a list of variable and parameter descriptions.

Each of the above models were developed using mathematical expressions that are derived from reasonable biological assumptions that we hypothesize can simulate the temporal dynamics observed in the *in vitro* data. To simulate combination therapies, the two single dose response models are combined into one system of three equations with 10 total parameters:

5 $$\frac{dT}{dt}=k{\textstyle (}1-f({A}_{b},t){\textstyle )}{\textstyle (}1-\frac{T}{\theta -h({P}_{i},t)}{\textstyle )}T$$

6 $$\frac{d{A}_{b}}{dt}={\textstyle \{}\begin{array}{cc}{\beta }_{A}({A}_{f}-{A}_{b})(T\cdot HER{2}_{exp}-{A}_{b}), & {A}_{f} > 0\\ 0, & {\rm{o}}{\rm{t}}{\rm{h}}{\rm{e}}{\rm{r}}{\rm{w}}{\rm{i}}{\rm{s}}{\rm{e}}\end{array}$$

7 $$\frac{d{P}_{i}}{dt}={\textstyle \{}\begin{array}{cc}{\alpha }_{P}({P}_{f}-{P}_{i})T, & {P}_{f} > 0\\ 0, & {\rm{o}}{\rm{t}}{\rm{h}}{\rm{e}}{\rm{r}}{\rm{w}}{\rm{i}}{\rm{s}}{\rm{e}}\end{array},$$

where *h*(*P*_*i*_, *t*) is now given by

$$h({P}_{i},t)={\delta }_{P}{\textstyle (}{e}^{-{\gamma }_{P}(t-{t}_{P}^{\ast })}+S{\textstyle )}{P}_{i},$$

and *S* is the “synergy” parameter between the two drugs. When paclitaxel is administered by itself, *S* = 1. In the following, other values for *S* will also be considered indicating a synergistic (*S* > 1) or non-synergistic (*S* < 1) effect of trastuzumab with paclitaxel. We note that there are other possible ways to mathematically characterize synergy between these drugs in this model system. Please see the Supplementary Materials for a comparison of five other forms of synergy we investigated including enhanced overall effect and timing of effect for both trastuzumab and paclitaxel as well as enhanced initial toxicity effects of paclitaxel. However, as none of these (more complex) formulations significantly improved the performance of the model in comparison to that characterized by Eq. (8), we only consider this formulation going forward.

Note that the model does not account for drug decay or loss over time for either therapy. This is a reasonable assumption as BT474 cells do not exhibit a significant level of efflux pumps for paclitaxel^17^. For trastuzumab, the media is changed before the half-life is reached (>1.7 days for breast cancer tissue^18^); therefore, the model need not account for drug loss. The complete removal of remaining free drug after 24 hours due to media changes was implemented in the numerical simulation.

The model was solved numerically using MATLAB’s (MathWorks, Natick, MA) ODE solver *ode45*, using a variable time step and initial conditions provided by the experimental data (MATLAB codes are available at https://github.com/OncologyModelingGroup/InVitroPacandTmAbSynergy). Confluences from the experimental data (see Cell Culture section) were converted to cell number using an average cellular radius of 1.25 × 10^−3^ *cm* and well area of 0.32 *cm*^2^ (measured using FIJI^19^).

### Parameter calibration

All of the parameters of the models were calibrated to the time-resolved microcopy data (apart from *HER2*_*exp*_, which is assigned based on the cell line^17,20,21^). The MATLAB function *fmincon* (an interior-point search algorithm) was used to find the optimal parameter values by minimizing the L2 norm of the weighted difference (to account for experimental error) between the model simulation and the mean of the data per time point (weighted by the 95% confidence intervals of the experimental data)—see Statistical Methods section for L2_err_ formula.

As the function *fmincon* is sensitive to the initial guess for the optimization search in the parameter space, for each dataset, a randomized vector of 100 initial guesses was used. The outcomes of these randomly seeded calibrations were compared to identify the parameter set with the lowest error between the resulting simulation and the data. Additionally, to limit the calibration parameter space, intervals for the growth rate and carrying capacity parameters (*k*, *θ*) were defined by calibrating the model to the control sets. Using larger ranges, *k* and *θ* were calibrated for all the control data. These control values were then used to define ranges for each of the two parameters for all the remaining, drug dosed sets—similar to other efforts using control data to define values for inherent parameters^22^.

**Figure 2:**
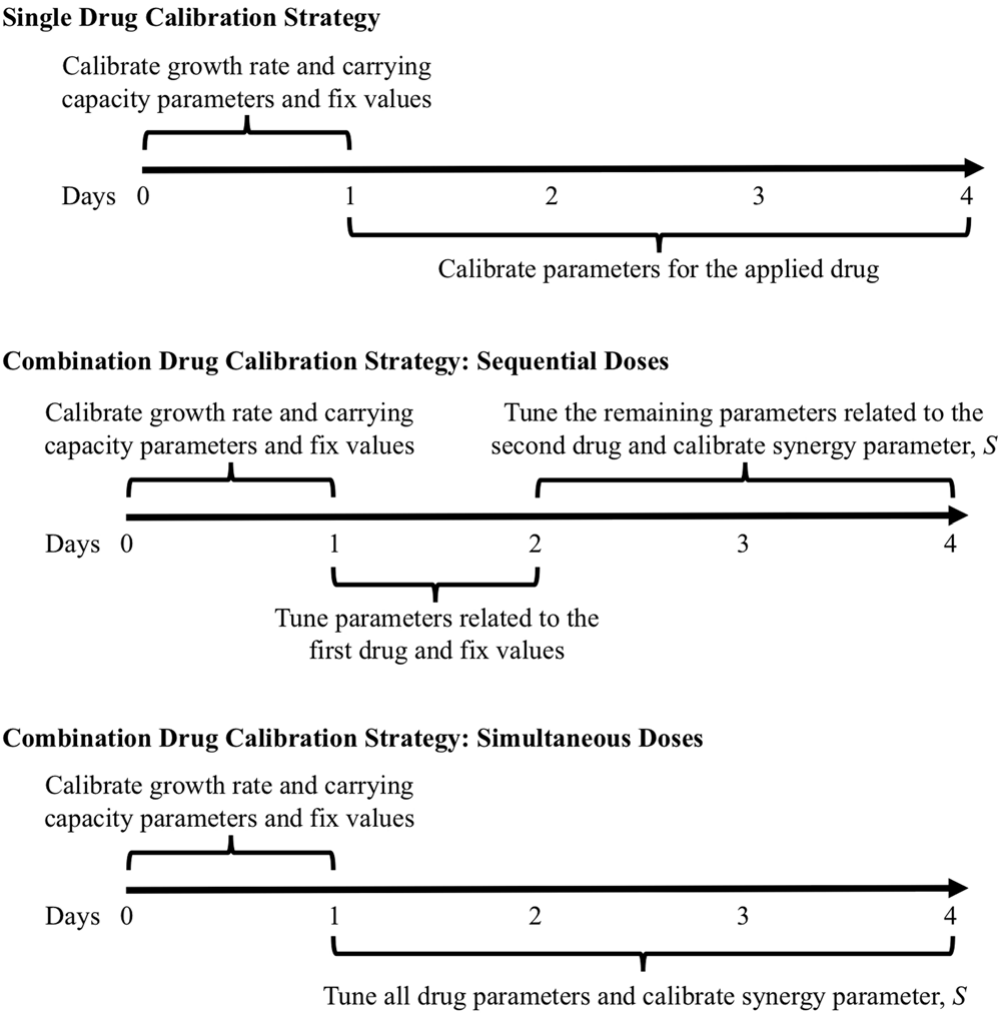
A schematic of the sequential calibration method for single drugs and combination drug regimens that administer the two drugs consecutively and simultaneously, as defined in the *in vitro* experimental timeline. For all calibration scenarios, parameters *k* and *θ* are calibrated using the first 24 hours of data within parameter intervals determined from the control data sets. For combination drug regimens, the apart from the synergy parameter, *S*, drug associated parameter undergo a small recalibration. This small recalibration is labeled as “tuning” because the parameter values were only allowed to vary within small, predetermined intervals about the calibrated values for the single drug dose sets for the paclitaxel and trastuzumab treatment. These intervals are defined using the calibration errors calculated for each parameter from the calibration verification study.

For the single drug models, parameter calibration was performed sequentially for different groups of parameters: pre-treatment and post-treatment parameters—see Fig. 2. For all cases, the first 24 hours where no drug was in the system (pre-treatment) was used to identify the native growth rate and carrying capacity (*k* and *θ*, respectively) of the cells using the predefined parameter ranges obtained from analyzing the control data. The parameters for the applied drug were determined with the remaining data following drug application (day 1 to 4). For example, for paclitaxel, the post-treatment parameters ${\delta }_{P},\,{\gamma }_{P},\,{t}_{P}^{\ast },\,{\alpha }_{P}$ are calibrated using the data points from days 1 to 4. Similarly, for trastuzumab the parameters ${\eta }_{A},\,{t}_{A}^{\ast },\,{\beta }_{A}$ are calibrated. This sequential calibration strategy limits the parameter space and generates parameter values for each drug where treatment parameters are not influenced by the native growth rate and carrying capacity.

A calibration verification study was performed for the sequential calibration (utilizing portions of the temporal data). Randomized *in silico* data was generated where the parameters were varied uniformly using a global sampling method (varying parameters simultaneously) to generate 100 randomized simulations for each of the single dose drug sets as well as controls with noise levels similar to those observed in the experimental data. The calibration method was applied to the *in silico* data to generate sets of optimized parameter groups that were then compared to the actual parameter values. Using all of the randomized sets, the average error to recover each parameter was calculated.

### Synergy for combination regimens in the mathematical model

First, the combined mathematical model was calibrated for growth and carrying capacity values using data acquired during the initial 24 hours. Then the model was simulated for the different combination regimens using the corresponding values derived from the single drug dose results to determine a baseline performance of the model where synergy was not considered. Then, to explore possible interaction effects between the two drugs, the parameter *S* was allowed to calibrate (from its nominal value of 1, which represents no synergy) for the combination regimens. The sequential calibration strategy, as described above for the single agent regimens was used; namely, the first 24 hours determined the initial growth rate and carrying capacity (*k* and *θ*, respectively) and are fixed, but *S* was calibrated using the data from day 2 to 4 (after both drugs are present)—see Fig. 2. Additionally, the data from day 1 to day 2 was used to perform a limited recalibration of the parameters associated with the first drug applied; specifically, instead of using the parameter values determined from the single dose calibration sets directly, the parameters corresponding to the first drug are restricted to vary within ranges defined by the mean calibration errors calculated for each parameter from the calibration verification study. The remaining parameters associated with the second drug were also recalibrated with the remaining data from day 2 to day 4. Similarly, for the combination regimens where the two drugs are given at the same time, the drug associated parameters were allowed to vary within their calibration error bounds plus the parameter *S* using the time resolved microscopy data from days 1 to 4 (see Fig. 2).

The parameters associated with the individual drugs were allowed to vary (within the calibration error bounds as described in the previous section from the single treatment experiments) to ensure that any values assigned to *S* (indicating any drug interaction relationships) could not simply be accounted for from small (error derived) variations in the parameter values. To reiterate, allowing parameters other than *S* to vary was an effort to capture values for *S* that would not be a “correction” value due to a poorer fit to the data resulting from using the fixed, single drug-dose derived parameters. With this strategy, the individual drug response data and combined model can be used to estimate *S* to quantify the synergy that may exist between paclitaxel and trastuzumab.

### Statistical methods

The mean and 95% confidence intervals for cell confluence for each *in vitro* group at each time point are reported, and a two-tailed t-test assessed differences between groups at the final time point (day 4). A p value of 0.05 or less was considered significant. The L2 norm of the weighted difference (to account for experimental error) between the model simulation and the mean of the data per time point (weighted by the 95% confidence intervals of the experimental data)

8 $${{\rm{L}}2}_{{\rm{e}}{\rm{r}}{\rm{r}}}=\sqrt{{\sum }_{t=0}^{{t}_{f}=4}{{\textstyle (}\frac{M(t)-O(t)}{Conf(t)}{\textstyle )}}^{2}}$$

where *M*(*t*) is the model simulation value at time *t*, *O*(*t*) is the observed value from the experimental data at time *t*, *Conf*(*t*) is the 95% confidence for the data at time *t*, and *t* is defined only for the experimental timepoints.

Further, the combination index (using the Loewe additivity null reference principle^23^) was calculated for the different regimens at day 4 to determine if the combined effects of the two drugs are synergistic, additive, or antagonistic:

9 $${\rm{combination}}\,{\rm{index}}=\frac{[{\rm{A}}]}{[{{\rm{A}}}^{\ast }]}+\frac{[{\rm{B}}]}{[{{\rm{B}}}^{\ast }]},$$

where [A] is the concentration of drug A for the combination regimen, and [A^*^] is the concentration of drug A for the monotherapy that achieves the same response; [B] and [B^*^] are similarly defined for the second drug. Loewe additivity indicates that two drugs are working synergistically, additively, or antagonistically if the combination index <1, =1, or >1, respectively^23^.

The concordance correlation coefficient (CCC) was calculated to compare the overall agreement in temporally dynamic trends of the model simulations to the *in vitro* data.

10 $${\rm{CCC}}=\frac{2\cdot CoV(x,y)}{Var(x)+Var(y)+{(\bar{x}-\bar{y})}^{2}}$$

where $\bar{x}$ is the means of the experimental data points, $\bar{y}$ is the values of the model simulation at each of the corresponding experimental time points of *x*, *Var* is the variance, and *CoV* the covariance.

## Results

### Treatment response

**Figure 3:**
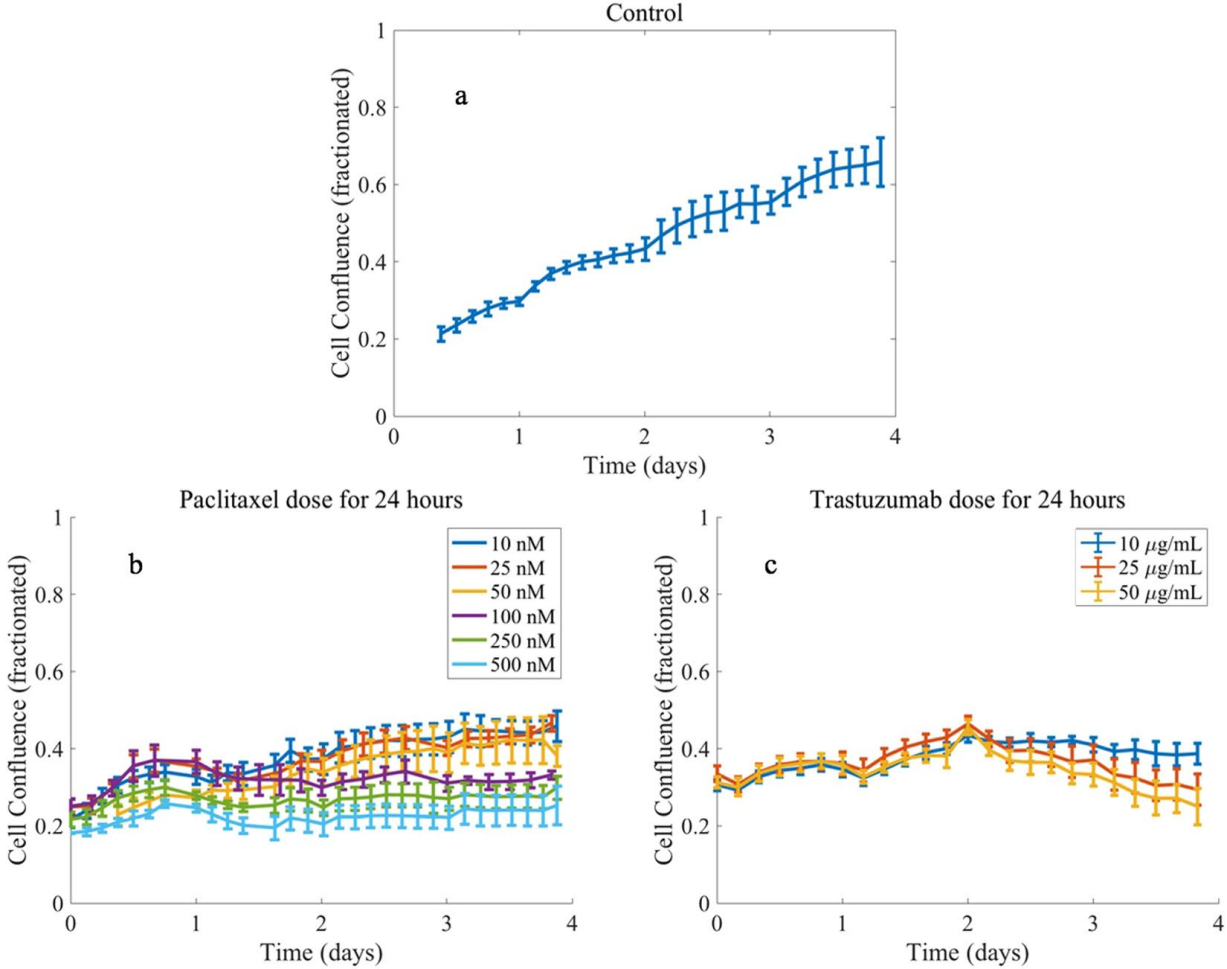
Comparison of one control (panel a) and varying drug doses for paclitaxel (panel b) and trastuzumab (panel c) administered individually. Data points are the mean of the data across replicates with 95% confidence intervals represented with error bars. Drugs are applied at day 1 and allowed to remain on the cells for 24 hours. After 24 hours the drug is removed, and media changed. Note that paclitaxel has an immediate effect on the tumor cell number compared to trastuzumab which had a delayed effect.

**Figure 4:**
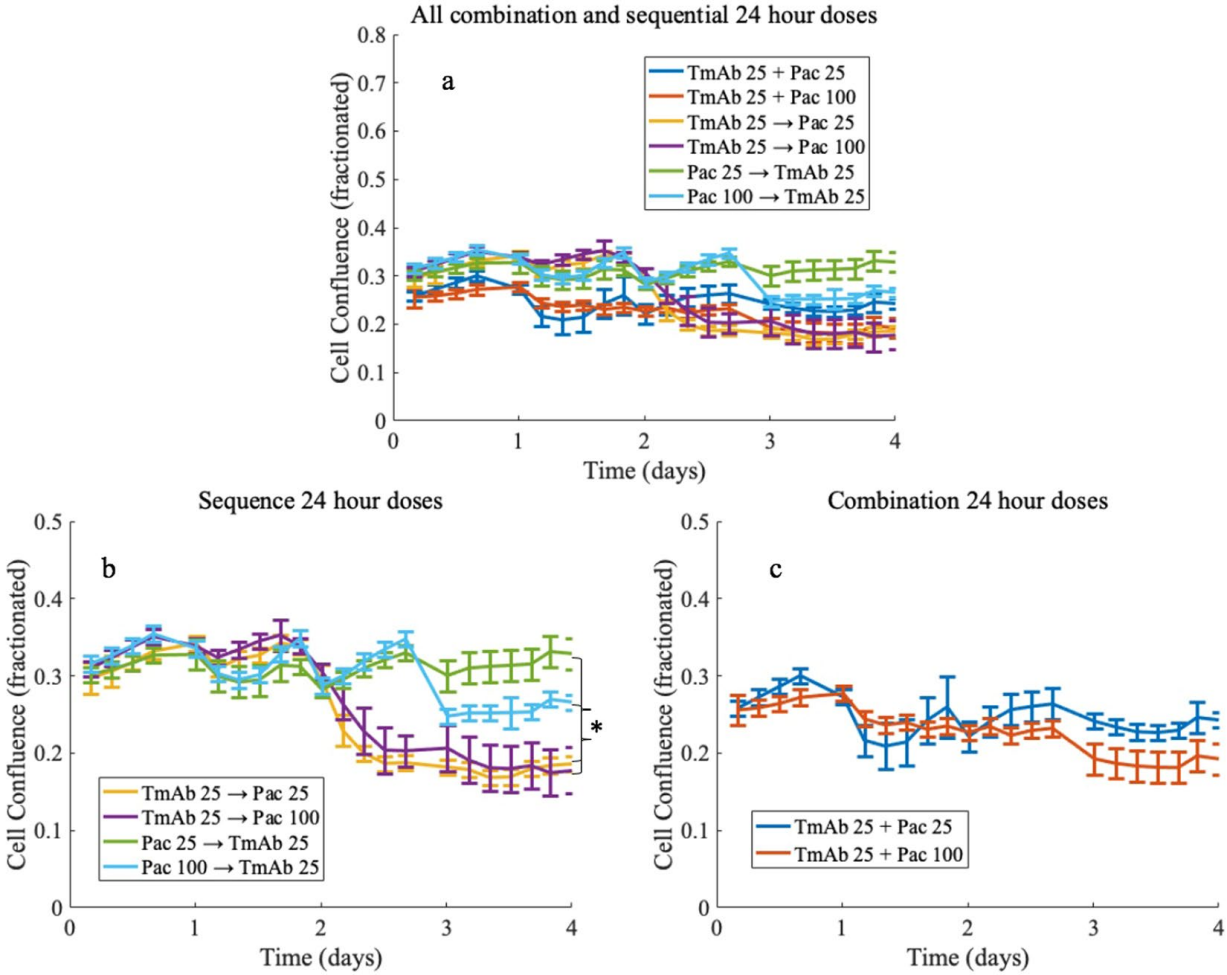
Comparison of combination regimens for two different dosages of paclitaxel (25 and 100 nM) with trastuzumab (panel a). Panel b depicts sequential combination regimens where either paclitaxel (Pac) or trastuzumab (TmAb, doses are *μ*g/mL) is applied first on day 1, then at day 2 the media is changed, and the second drug is applied; finally, after 24 hours (day 3) the second drug is also removed, and the media changed. Note the significant difference by day 4 between the sequences where paclitaxel is administered first versus trastuzumab first. Panel c shows the results for the drugs applied simultaneously for 24 hours (day 1 to day 2) for two different paclitaxel doses.

Compared to controls, treatment with paclitaxel or trastuzumab monotherapy results in significantly lower tumor cell numbers by day 4, independent of the dose administered (p < 0.001)—see Table 3 for the resulting confluence values and their 95% confidence intervals for each dose at the final time. Figure 3 illustrates cell growth during the first 24 hours prior to drug administration, followed by a dose-dependent treatment response. In Fig. 4, significant differences are observed between the different therapeutic regimens, where sequences that administered trastuzumab first result in lower recorded confluences at day 4 compared to paclitaxel first, independent of dose administered (p < 0.001)—see Table 4 for the resulting mean confluences and their 95% confidence intervals at day 4 for each of the combination doses. Table 5 lists the combination index of each of the combination regimens. The combination regimens where paclitaxel is given prior to trastuzumab trend toward an additive effect according to the combination index results, whereas administering trastuzumab first results in synergy between the two drugs (with combination index values less than 0.7). None of the drug combinations tested resulted in a combination index indicating antagonism. Nonspecific changes in the data as seen by small abrupt oscillations in cell confluence are noted to occur during periods of media washes (to remove therapeutics or change media) and interferences in scheduled timings for the Incucyte system (managing six separate plates).

**Table 3:** Day 4 mean confluence (fractionated) and 95% confidence intervals for single drug regimens across all doses evaluated. For controls mean fractionated confluence is 0.61 with confidence interval [0.55, 0.67].

Dosage	Paclitaxel dose (nM) result		Trastuzumab dose (*μ*g/mL) result	
	Mean confluence (fractionated)	95% confidence	Mean confluence (fractionated)	95% confidence
10	0.45	[0.41, 0.49]	0.43	[0.42, 0.45]
25	0.46	[0.43, 0.49]	0.38	[0.36, 0.40]
50	0.38	[0.35, 0.40]	0.24	[0.21, 0.27]
100	0.33	[0.32, 0.34]		
250	0.30	[0.27, 0.33]		
500	0.25	[0.21, 0.29]

**Table 4:** Day 4 mean confluence (fractionated) and 95% confidence intervals for combination drug regimens for two different paclitaxel dosages.

Regimen	Paclitaxel 25 nM, Trastuzumab 25 *μ*g/mL		Paclitaxel 100 nM, Trastuzumab 25 *μ*g/mL	
	Mean confluence (fractionated)	95% confidence	Mean confluence (fractionated)	95% confidence
Paclitaxel prior to trastuzumab	0.33	[0.31, 0.35]	0.27	[0.26, 0.28]
Paclitaxel and trastuzumab applied simultaneously	0.24	[0.23, 0.25]	0.19	[0.17, 0.21]
Trastuzumab prior to paclitaxel	0.19	[0.18, 0.20]	0.18	[0.15, 0.21]

**Table 5:** Calibrated synergy values for parameter *S* and approximated combination index values for the different combination therapy experiments.

Regimen	Paclitaxel 25 nM, Trastuzumab 25 *μ*g/mL		Paclitaxel 100 nM, Trastuzumab 25 *μ*g/mL	
	*S*	Combination Index	*S*	Combination Index
Paclitaxel prior to trastuzumab	0.09	0.75	0.71	[0.7, 0.9]
Paclitaxel and trastuzumab applied simultaneously	0.13	0.55	1.22	<0.7
Trastuzumab prior to paclitaxel	1.47	<0.55	1.24	<0.7

### Model fits for controls and single therapy regimens

**Figure 5:**
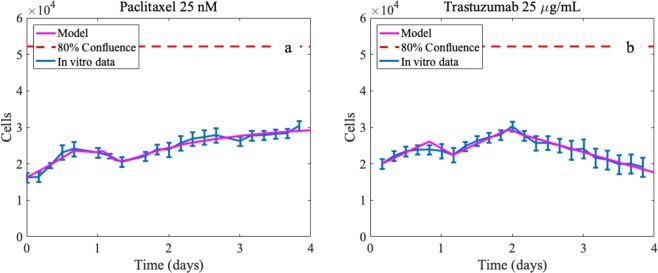
Example fits for the mathematical model after being calibrated to the individual drug doses for paclitaxel and trastuzumab. (Panel a) paclitaxel 25 nM with CCC = 0.98 and (**b**) trastuzumab 25 *μ*g/mL with CCC = 0.96 between the model simulation and the data. Note that the experimental confluence values are converted to approximate number of tumor cells to simulate the model.

Equations (1–4) were calibrated to each of the single dose regimens and controls. The median parameter error from the calibration method is 0.83% across all parameters (maximum of 2.7% and minimum of 0.2%). Figure 5 illustrates example model fits for one paclitaxel and trastuzumab treated experimental set. Please see the Supplementary Materials (Tables S1–3 for all individual CCC and L2_err_ values for each experimental data set as well as all of the calibrated parameter sets and their associated errors.

### Modeling combination therapies and synergistic effects

**Figure 6:**
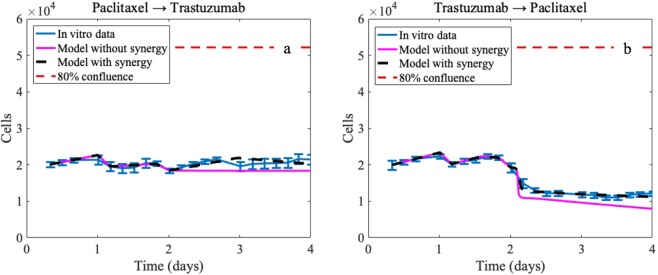
Example simulations of the mathematical model for two different combination regimens (both with paclitaxel 25 nM and trastuzumab 25 *μ*g/mL). Panel (a) corresponds to results for the paclitaxel added prior to trastuzumab experimental sets, and panel (b) corresponds to results for the trastuzumab added prior to paclitaxel experimental sets. Panel (a) shows the results for the model simulation where the parameters from the single drug doses are used explicitly and synergy is not considered (CCC = 0.23) as well as the results for the model simulation where the synergy parameter is included in the recalibration (CCC = 0.61). Similarly, for panel (b), the results for the model simulation where the parameters from the single drug doses are used explicitly CCC = 0.94, whereas the results for the model simulation where the synergy parameter is included in the recalibration CCC = 0.99. Note that the experimental confluence values are converted to approximate number of tumor cells to simulate the model.

Using the parameters derived from the single dose regimens for the combination therapies summarized in Table 1, model simulations did not result in good matches (based on CCC values) between the predictions and the *in vitro* data across all the different combination regimens. Comparing these simulations to the each of the experimental data sets, the median CCC = 0.42 with range [0.01, 0.94]. Overall, calibration of the model including the synergy parameter *S*, improves the median CCC value 0.88 with range [0.61, 0.99] (compared to not including *S* which results in median CCC = 0.78 range of [0.16, 0.99]). Please see the Supplementary Materials (Tables S4, S7) for all individual CCC and L2_err_ values for each experimental data set. Figure 6 presents examples of the model’s simulations compared to the experimental data, where simulations including the synergy parameter have better agreement with the data than simulations without the synergy parameter.

Table 5 compares the calibrated values of *S* for each of the combination therapy experiments. For both dosage levels of the treatment sequencing experiments, if paclitaxel is administered first, then 0 < *S* < 1, whereas if trastuzumab is administered first, *S* > 1. Further, the results of the combination therapy doses (where the two drugs are given at the same time), the *S* value lies inside the intervals defined by the paclitaxel first versus trastuzumab first values. Quantitative assessment for other possible synergistic parameters effects assessed for this mathematical model are in the Supplementary Materials.

## Discussion

The sequencing of paclitaxel and trastuzumab combinations has a significant effect on the treatment response and dynamic changes in cell confluence for the BT474, HER2+ breast cancer cells. The data indicates that the two drugs can function both additively and synergistically depending on how they are administered for this cell line. Specifically, the dosage and timing of the two drugs governs the level of overall synergism that can be observed. In particular, regimens where trastuzumab was applied first followed by paclitaxel had the greatest overall reduction in tumor cells compared to the opposite ordering. Calculation of the combination index shows the change in synergism quantitatively with paclitaxel first resulting in only a trend toward an additive effect. As these therapies are traditionally administered simultaneously for standard-of-care and several other groups have evaluated the combination of trastuzumab and paclitaxel administered simultaneously for enhanced response and toxicity^24–26^, these results suggest that sequencing should be explored further in *in vivo* studies and that simultaneous delivery of the two drugs may not be optimal. Also, note that the 24 hr drug application used here is more biologically relevant compared to other *in vitro* assays where the drug is left on the cells for the entirety of the experiment^24–26^.

The mathematical models, developed with biologically relevant assumptions and an integrated experimental-computational approach, were able to faithfully simulate the dynamics of the *in vitro* results. The calibration method was verified with *in silico* generated data indicating that parameter recovery errors are small, and the resulting model simulation fits for the single drug dose sets showed good agreement to the data as determined with CCC values. When analyzed with the combination data sets, a combined mathematical model offered another avenue of investigation for the synergy between the two drugs. Several different synergism parameters were explored, but only *S*—the parameter altering the overall effectiveness of paclitaxel treatment—offered the greatest overall improvement in the fit to the data (see the Supplementary Materials for those results). Notably, modifications to trastuzumab effect/binding, changes in delay/timing parameters of the two drugs, and paclitaxel uptake/toxicity did not result in calibrations that dramatically outperformed those with *S* alone. This does not suggest that these other interactions are not present or do not have an effect on the two drugs’ synergy; however, for the data presented here, the model and analysis narrow the scope of our investigation to *S* as the most fruitful, initial path for study.

Changes in the synergy parameter (*S*) directly alter the end behavior of the potential overall effect that paclitaxel can reduce the carrying capacity in the mathematical model as time progresses. As this parameter is nominally set to *S* = 1 for paclitaxel alone dosing, resulting calibration values from a combination/sequence set where 0 < *S* < 1 suggests there may be additivity or antagonism between the two drugs that reduce the overall potential effect paclitaxel can have on the carrying capacity. Values of *S* > 1, suggest an enhancement of paclitaxel’s ability to reduce the carrying capacity—indicating synergy between the two drugs. The synergy parameter also demonstrates how the ordering of the two drugs can increase and decrease the synergy for different combination regimens—suggesting that there exists a spectrum of synergistic effects dependent on the ordering of the two drugs. Also note, that this effect is more pronounced in the lower paclitaxel dose. In the mathematical model, *S* governing the end behavior of paclitaxel’s effect is biologically related to and can be interpreted as the drugs ability to terminally arrest the division of cells. It has become better understood that paclitaxel does not always (nor does it often) cause a cell to immediately arrest during mitosis but, instead, causes abnormal divisions^6^. These abnormal divisions subsequently result in impassable growth checkpoints for the cell and ultimately death. Therefore, changes in the *S* parameter correspond to changes in paclitaxel’s speed in inducing cellular arrest and more terminally prevent future cellular divisions. Considering how the ordering of the drugs resulted in different responses by the cells and the corresponding values of *S* (derived directly from the data), it appears that trastuzumab being administered first primes or sensitizes the cells in a manner that enhances paclitaxel’s long-term effect—perhaps due to the intra-cellular signaling cascade caused by trastuzumab binding to HER2.

While the two synergy measures agree (the combination index and the calibrated values of *S*), only the results from the mechanistic mathematical modeling are hypothesis generating in regard to the biological phenomena behind this effect. Many studies have focused on the quantification and definition of synergistic effects of drugs^1,23,27–30^, and the results of these studies are useful for identifying the presence and measuring the amount of synergy between drugs. However, the results cannot be used to help understand the molecular/cellular drivers of synergy because expressions for drug mechanisms have not been incorporated. Our work utilizes *in vitro* cellular scale data to describe the synergism between the two drugs as well as elicit information about the biological cause of this synergy—biologically identifying mechanisms of drug synergy is difficult and data-driven. Mathematical models can be used to recognize potential cellular mechanisms from where synergy might be derived. Other mechanistic modeling efforts for synergy of anti-cancer therapeutics have directly considered signaling networks with kinetic, partial differential equation, agent-based, and even multi-scale models^31–34^. However, these studies are limited by their computational expensive and data demanding nature (requiring large amounts of time course proteomics and/or transcriptomics) and do not consider synergism dependent on the sequencing or timing of drugs.

Limitations of the current study include the use of one cell line and focused drug doses. Additional *in vitro* data is important to better characterize the temporal dependence of the synergy between the two drugs by looking at more combinations. Moreover, this should be investigated in multiple HER2+ cell lines to define ranges of the effectiveness of the two drugs for varying levels of HER2 expression. Due to the fact that patient-to-patient variability of synergistic, additive, or antagonistic effect response is likely due to the diverse genetic population^35^ the response of various HER2+ cell lines must be characterized to design the model to have the ability to handle heterogeneity. More *in vitro* data is required to better characterize the synergy between the two drugs if these drug regimens are to be optimized. The mathematical model introduced here could be used to generate optimal therapy combinations (testing limitless paclitaxel-trastuzumab regimens *in silico*). However, results from additional dosing regimens and timings of drug combinations would both guide and restrict the model to have more accurate predictions and parameter estimates by leveraging additional data. Further, with additional data, detailed uncertainty quantification studies comparing the model simulations to the data are merited. With the uncertainty results and a larger parameter space to explore, variance-based global sensitivity analysis techniques can be applied to determine any potential interacting effects of parameters that may affect calibration results. Also, data relating to the uptake and binding of paclitaxel and trastuzumab, respectively, will further inform the mathematical model’s calibrations reducing the uncertainty in the results. Finally, this work is based on an *in vitro* experimental design, and future work will be needed to validate these synergistic dynamics *in vivo* to include (for example) interactions between the tumor cells and the microenvironment for translational impact^15,36^.

The *in vitro* evidence presented here provides a justification for future study of the interactions and temporal dependencies of cytotoxic and targeted drug synergy. Additionally, with a mathematical model we can begin to explore the intricacies of trastuzumab-paclitaxel combination effects and the enumerable potential regimens for the two drugs. We have presented a simple example of how experimental investigations supported by mathematical models can generate experimentally-testable hypotheses, and by fortifying such a framework, a mathematical model can eventually be used to optimize regimens for combination therapy. Furthermore, a future direction of this work is to couple the cellular scale model presented here to a tissue scale mathematical model—developed from *in vivo* pre-clinical studies in mice^36^—to leverage the *in vitro* data to predict the outcomes of combination therapy *in vivo*. This is an important step for future clinical translation to provide temporal guidance of standard-of-care, combination therapies—potentially leading to significantly improved anticancer response in HER2+ breast cancer.

## Supplementary information

Supplemental Materials

## Data Availability

The datasets generated during and/or analyzed during the current study are available from the corresponding author on reasonable request.
