# Supplementary material for: Experimentally-driven mathematical modeling to improve combination targeted and cytotoxic therapy for HER2+ breast cancer
Source: Sci Rep. 2019 Sep 6;9:12830. doi: 10.1038/s41598-019-49073-5 (PMC6731321; doi:10.1038/s41598-019-49073-5)
Supplement: Supplementary file 1 — Supplemental Materials [file 41598_2019_49073_MOESM1_ESM.pdf]

Experimentally-driven mathematical modeling to improve combination targeted and cytotoxic therapy for HER2+ breast cancer

Angela M. Jarrett[1,2], Alay Shah[3], Meghan J. Bloom[3], Matthew T. McKenna[6], David A. Hormuth[1,2], II, *Thomas E. Yankeelov[1-5], *Anna G. Sorace[7-9]

[1]Institute for Computational Engineering and Sciences
[2]Livestrong Cancer Institutes
[3]Department of Biomedical Engineering
[4]Department of Diagnostic Medicine
[5]Department of Oncology
The University of Texas at Austin
Austin, Texas, USA

[6]Department of Biomedical Engineering
Vanderbilt University
Nashville, Tennessee 37232

[7]Department of Radiology
[8]Department of Biomedical Engineering
[9]O'Neal Comprehensive Cancer Center
University of Alabama at Birmingham
Birmingham, AL 35209

*indicates correspondence

Please address correspondence to:

Anna G. Sorace, Ph.D.
University of Alabama at Birmingham
Birmingham, AL 35233
asorace@uabmc.edu

Thomas E. Yankeelov, Ph.D.
The University of Texas at Austin
Austin, Texas 78712
thomas.yankeelov@utexas.edu

Supplemental Materials

Calibration results for controls and single dose sets

Tables S.1-3 summarize the calibration results for all controls and single drug dose sets. Reported are the parameter values, CCC and $L2_{err}$ quantifying the fits to the data, and the calibration recovery error percentages for each parameter.

Table S.1: Resulting calibration values for each of the control sets—including the parameter recovery errors for the growth and carrying capacity using only the first 24 hours of data to determine their values.

Control Set	k	θ	CCC	$L2_{err}$
**1**	0.36	0.91	0.99	5.18
**2**	0.95	0.68	0.84	3.45
**3**	0.92	0.67	0.77	6.59
**4**	1.12	0.59	0.90	5.50
**5**	0.83	0.44	0.65	2.04
**6**	0.53	0.74	0.96	4.40
**7**	0.64	0.66	0.96	4.20
**Median**	0.83	0.67	0.90	4.40
**Minimum**	0.36	0.44	0.65	2.04
**Maximum**	1.12	0.91	0.99	6.59
**Mean Parameter Recovery Error**	0.90%	0.32%		

Table S.2: Resulting calibration values for each of the paclitaxel single dose sets—including the parameter recovery errors for the paclitaxel associated parameters.

Paclitaxel Dose	k	θ	δ_P	γ_P	t_P^*	α_P	CCC	$L2_{err}$
**10 nM**	1.12	0.72	$1.36\text{x}10^2$	9.63	1.38	0.75	0.99	1.60
**25 nM**	0.84	0.91	$0.93\text{x}10^2$	6.68	1.48	0.67	0.98	1.95
**50 nM**	1.12	0.49	$0.08\text{x}10^2$	6.03	1.58	0.73	0.98	1.56
**100 nM**	0.82	0.91	$0.35\text{x}10^2$	5.43	1.46	0.66	0.89	2.01
**250 nM**	1.12	0.45	$0.04\text{x}10^2$	6.67	1.48	0.68	0.93	1.51
**500 nM**	0.54	0.91	$0.12\text{x}10^2$	5.37	1.49	0.58	0.93	1.25
**Mean Parameter Recovery Error**			0.38%	1.08%	0.21%	1.28%		

Table S.3: Resulting calibration values for each of the trastuzumab single dose sets—including the parameter recovery errors for the trastuzumab associated parameters.

Trastuzumab Dose	k	θ	η_A	t_A^*	β_A	CCC	$L2_{err}$
**10 μg/mL**	0.69	0.91	4.29	1.80	9.26	0.96	2.30
**25 μg/mL**	0.79	0.71	2.51	1.99	8.78	0.96	1.59
**50 μg/mL**	0.77	0.80	1.82	1.93	6.62	0.94	2.76
**Mean Parameter Recovery Error**			0.83%	0.30%	2.71%		

Alternative synergy parameter exploration

In addition to the S parameter in the main text, five other possible synergistic effects were assessed in the mathematical model. Here the synergy parameter above, S is relabeled as S_1. **Equation (S.1)** shows all the synergy parameter possibilities that were tested together in the tumor cell response equation (1), where functions $f(A_b,t)$ and $h(P_i,t)$ are directly incorporating into the equation:

$$\frac{dT}{dt} = k\left(1 - S_6\eta_A A_b(t > S_5 t_A^*)\right)\left(1 - \frac{T}{\theta - S_4\delta_P\left(e^{-S_3\kappa\left(t - S_2 t_P^*\right)} + S_1\right)P_i}\right)T \quad \text{(S.3)}$$

Note that for all of these synergy parameters (S_1 - S_6), their nominal values are equal to one. For each of these parameters, the model was recalibrated allowing the base parameters to vary within their corresponding attributable calibration errors along with each synergy parameter (one at a time). In **Table S.4**, a summary of all the calibration results is reported. Note that, across all the synergy parameter possibilities, S_1 and S_4 result in the greatest reduction in the $L2_{err}$ values on average for the different sequence sets—with S_1 having the best resulting fits. The parameter S_1 will only govern the end behavior for the effect of paclitaxel over time, where greater or lesser values of S_1 will result in greater and lesser reductions in the overall cellular carrying capacity, respectively. Similarly, the parameter S_4 adjusts the overall effect of paclitaxel—both its initial toxicity and end behavior—where, again, greater or lesser values of S_4 will result in greater and lesser reductions in the overall cellular carrying capacity, respectively. Looking at the resulting calibration values for each of the synergy parameters (**Table S.5**), a pattern emerges for paclitaxel first versus trastuzumab first sets; greater synergy or enhancement of the efficacy of the two drugs together increases with trastuzumab being administered first. Additionally, a model calibration was performed where both synergy parameters were included (S_1 and S_4 together) to determine if the combined degrees of freedom could further improve the simulation fits to the data (**Table S.6**),

but incorporating both parameters did not improve the results beyond that of S_1 alone. Therefore, to have the most parsimonious model while also being able to represent the experimental data, only S_1 was considered for the combination dose sets (two drugs given at the same time). **Table S.7** summarizes the calibration results for each of the combination sets where trastuzumab and paclitaxel are applied to the cells at the same time.

Table S.4: Resulting calibration results for each of the sequence combination sets where either paclitaxel (Pac) or trastuzumab (TmAb) was administered prior to the other drug. Summarized are the CCC and $L2_{err}$ values for the model simulation compared to the means of the data using the previously calibrated values from the single dose set (which we label as "Fixed"), when only the base parameters are allowed to vary within their attributable calibration error bounds (labeled "Error Variation"), and for the resulting simulation fits where each synergy parameter (S_1-S_6) is also included.

**Dosage**		**Trastuzumab 25 μg/mL, Paclitaxel 25 nM**		**Trastuzumab 25 μg/mL, Paclitaxel 100 nM**		**Mean**
**Sequence**		**TmAb → Pac**	**Pac → TmAb**	**TmAb → Pac**	**Pac → TmAb**	
**Fixed**	**CCC**	0.94	0.23	0.92	0.36	0.61
	**$L2_{err}$**	11.57	7.66	4.02	29.66	13.23
**Error Variation**	**CCC**	0.99	0.16	0.99	0.42	0.64
	**$L2_{err}$**	2.98	8.07	1.27	29.42	10.43
S_1	**CCC**	0.99	0.61	0.99	0.62	0.80
	**$L2_{err}$**	2.33	2.94	0.85	10.76	4.22
S_2	**CCC**	0.99	0.16	0.99	0.50	0.64
	**$L2_{err}$**	3.06	8.01	1.27	21.29	10.40
S_3	**CCC**	0.99	0.16	0.99	0.42	0.64
	**$L2_{err}$**	2.94	8.01	1.27	29.25	10.37
S_4	**CCC**	0.98	0.61	0.99	0.62	0.80
	**$L2_{err}$**	5.89	2.94	1.15	10.76	5.18
S_5	**CCC**	0.99	0.16	0.99	0.50	0.66
	**$L2_{err}$**	3.02	8.01	1.27	21.29	8.40
S_6	**CCC**	0.99	0.16	0.99	0.42	0.64
	**$L2_{err}$**	2.61	8.06	0.76	29.43	10.21

Table S.5: Resulting calibration values for S_1 and S_4 from fitting the sequence combination sets where either paclitaxel (Pac) or trastuzumab (TmAb) was administered prior to the other drug. Note that the two parameters reveal the same pattern where greater synergy/enhancement is seen for sequences where trastuzumab is given prior to paclitaxel. Whereas for the paclitaxel first sets, these parameters have reduced values (from their nominal values of one).

**Dosage**	**Trastuzumab 25 μg/mL, Paclitaxel 25 nM**	**Trastuzumab 25 μg/mL, Paclitaxel 100 nM**

Sequence	TmAb → Pac	Pac → TmAb	TmAb → Pac	Pac → TmAb
S_1	1.47	0.09	1.24	0.71
S_4	1.04	0.10	1.14	0.71

Table S.6: Resulting calibration results for S_1 and S_4 together from fitting the sequence combination sets where either paclitaxel (Pac) or trastuzumab (TmAb) was administered prior to the other drug. Note that combining the two parameters together does not greatly improve the overall fits from that of S_1 by itself (see **Table S.4**).

Dosage		Trastuzumab 25 μg/mL, Paclitaxel 25 nM		Trastuzumab 25 μg/mL, Paclitaxel 100 nM	
Sequence		TmAb → Pac	Pac → TmAb	TmAb → Pac	Pac → TmAb
$S_1 + S_4$	CCC	0.99	0.61	0.99	0.62
	$L2_{err}$	2.34	2.94	1.01	10.75

Table S.7: Resulting calibration results from fitting the combination sets where paclitaxel (Pac) and trastuzumab (TmAb) were administered at the same time. Summarized are the CCC and $L2_{err}$ values for the model simulation compared to the means of the data using the previously calibrated values from the single dose set (which we label as "Fixed"), when only the base parameters are allowed to vary within their attributable calibration error bounds (labeled "Error Variation"), and for the resulting simulation fits where the S_1 synergy parameter is included.

Dosage		Trastuzumab 25 μg/mL, Paclitaxel 25 nM	Trastuzumab 25 μg/mL, Paclitaxel 100 nM
Fixed	CCC	0.01	0.47
	$L2_{err}$	17.95	8.45
Error Variation	CCC	0.87	0.70
	$L2_{err}$	2.60	5.14
S_1	CCC	0.87	0.90
	$L2_{err}$	2.58	4.28
